# Inhibition of TRPM7 Channels Reduces Degranulation and Release of Cytokines in Rat Bone Marrow-Derived Mast Cells

**DOI:** 10.3390/ijms150711817

**Published:** 2014-07-03

**Authors:** Linjie Huang, Ngai-Mui Ng, Ming Chen, Xiaoling Lin, Tiantian Tang, Huihua Cheng, Cheng Yang, Shanping Jiang

**Affiliations:** 1 Department of Respiratory Medicine, Sun Yat-sen Memorial Hospital, Institute for Respiratory Disease of Sun Yat-sen University, Sun Yat-sen University, Guangzhou 510120, China; E-Mails: hlinj@mail.sysu.edu.cn (L.H.); chenmingsysu@gmail.com (M.C.); xiaolinglinsysu@gmail.com (X.L.); tiantiantangsysu@gmail.com (T.T.); huihuachengsysu@gmail.com (H.C.); 2 Clinical Department of School of Medicine, Shenzhen University, Shenzhen 518060, China; E-Mail: wuyimei@szu.edu.cn; 3 Department of Respiratory Medicine, Mei Zhou People’s Hospital, Meizhou Affiliated Hospital of Sun Yat-sen University, Meizhou 514031, China; E-Mail: yangchengsysu@gmail.com

**Keywords:** rat, asthma, bone marrow-derived mast cells (BMMCs), transient receptor potential melastatin 7 (TRPM7), 2-aminoethoxydiphenyl borate (2-APB), shRNA, cytokines

## Abstract

Background: mast cells play an important role in airway inflammation in asthma. The transient receptor potential melastatin-like 7 (TRPM7) channel is expressed in primary human lung mast cells and plays a critical role for cell survival. This study aimed to investigate the role of TRPM7 on degranulation and release of cytokines in rat bone marrow-derived mast cells (BMMCs). Methods: the expression levels of TRPM7 were observed by immunocytochemistry and RT-PCR between normal and asthmatic rat BMMCs. TRPM7-specific shRNA and 2-aminoethoxydiphenyl borate (2-APB) and specific shTRPM7 were used to inhibit the function of TRPM7. Degranulation levels were analyzed by beta-hexosaminidase assay. Histamine, TNF-α, IL-6 and IL-13 levels were measured by ELISA. Results: the expression of TRPM7 was significantly higher in asthmatic rat BMMCs than in the normal control group. After application of 2-APB and down-regulation of TRPM7, the beta-hexosaminidase activity and secretion of histamine, IL-6, IL-13 and TNF-α were significantly decreased in the asthmatic group compared to the control group. Conclusion: this study indicates that TRPM7 channels may be involved in the process of degranulation and release of cytokines in rat bone marrow-derived mast cells.

## 1. Introduction

Asthma is characterized by chronic inflammation and infiltration of the mucosa with mast cells, eosinophils, lymphocytes and other inflammatory corpuscles [1]. Activated mucosal mast cells release the major pulmonary source of histamine and other inflammatory mediators such as TNF-α, IL-6 and IL-13 levels [2]. Increased mast cells in airway smooth muscle could play a key role in the process of airway hyperresponsiveness in asthma [3], and patients with atopic uncontrolled asthma have an increased parenchymal infiltration of mast cells compared with a control group [4].

In mast cells, calcium channel mobilization is essential to many cellular processes, including stimulated exocytosis and cytokine production [5]. Activation of mast cells by allergens has a critical connection with the influx of Ca^2+^ from the extracellular space [6,7]. As mast cells are nonexcitable cells, the main routes of Ca^2+^ entry are store-operated channels or Ca^2+^-permeable non-selective cation channels [8]. Although significant progress has been made in the characterization of Ca^2+^ entry on mast cells, the precise function of individual channels is largely unknown.

The transient receptor potential melastatin 7 (TRPM7) is a bifunctional protein characterized by ion channel and kinase activity [9]. The notable feature of TRPM7 is the permeation of Ca^2+^, Mg^2+^, and trace metals, suggesting its possible role for calcium entry [10]. The expression of TRPM7 is found in all examined mast cell types [11], including primary human lung mast cells, human cell line LAD-2 and HMC-1 [12], as well as RBL-2H3 cell line [13]. Recently, research has shown that TRPM7 plays a critical role for primary lung mast cell survival [12]. Therefore, TRPM7 may be an important channel for mast cells.

Rat bone marrow-derived mast cells (BMMCs) possesses phenotypic characteristics of mucosal mast cells [14]. In this study, we investigated the expression of TRPM7 on rat BMMCs, and its possible role in degranulation and release of cytokines after antigen stimulation. The function of TRPM7 was inhibited by two means, including the pharmacological blockade by 2-aminoethoxydiphenyl borate (2-APB), and the application of a lentiviral shRNA delivery system.

## 2. Results

### 2.1. Light Microscopy of Rat Bone Marrow-Derived Mast Cells (BMMCs)

**Figure 1:**
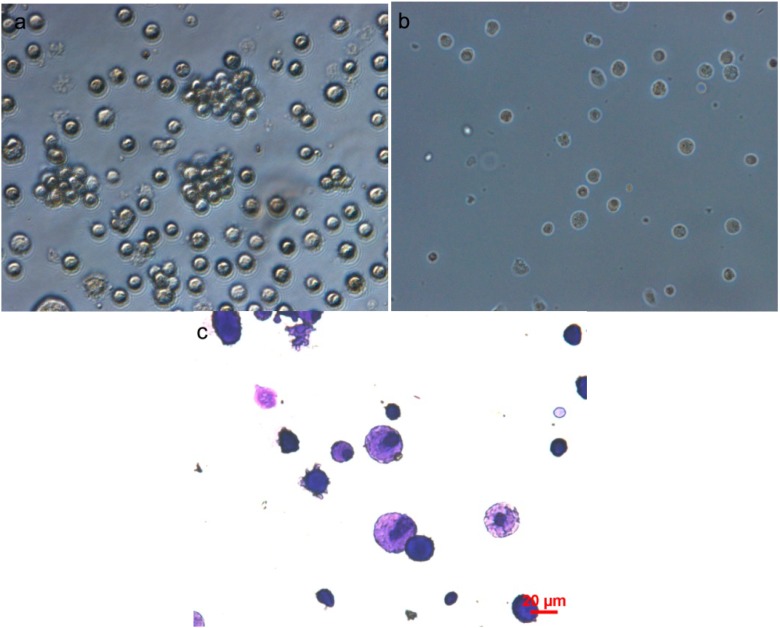
Light microscopic of rat bone marrow-derived mast cells (BMMCs). (**a**) BMMCs were diverse in size and had blast like structures in cell clusters; (**b**) Some of the cells were differentiated into adherent cells; (**c**) Cells stained by Toluidine blue. Scale bar in (**c**) can represent the ones for (**a**) and (**b**).

Rat BMMCs cultured in rrIL-3 were visualized by light microscopy. Day 28 cells (Figure 1a) were diverse in size and had blast like structures in cell clusters, some of the cells can be differentiated into adherent cells (Figure 1b). When stained by Toluidine blue, 28-day cells (Figure 1c) had pinkish cytoplasmic staining.

### 2.2. Flow Cytometric Analysis of Rat BMMCs

**Figure 2:**
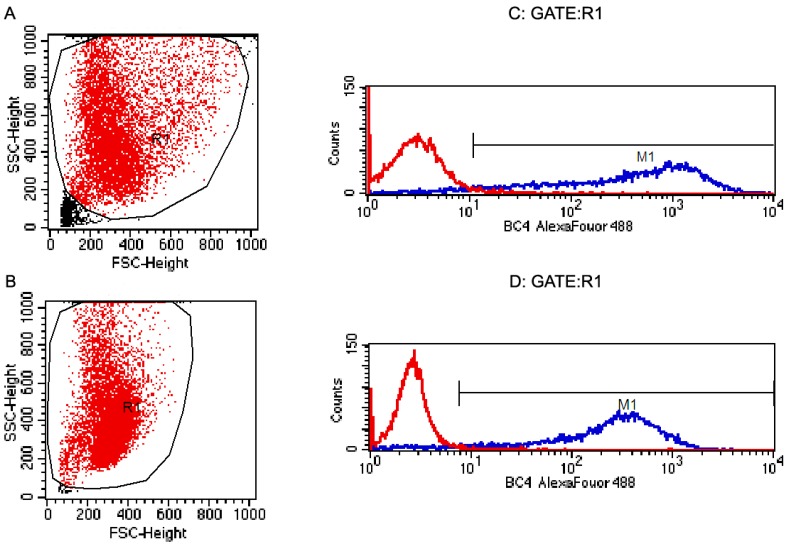
Flow cytometry of the surface expression of on rat BMMCs. Cell were analyzed by flow cytometry for cell size by both FSC and SSC (**A**, **B**); and for surface expression of the BC4 (**C**, **D**) antigen. Controls with red line in (**C**, **D**) were only treated with the secondary antibody. The blue lines in (**C**, **D**) were for the membrane protein for the primary antibody, BC4, and the 2nd antibody. High fluorescent events have very high BC4 (FcεRI) fluorescence and their distribution in BMMCs. These confirm that more than 95% of the cells were BC4 positive. Normal rat BMMCs (**A** ,**C**); Asthmatic rat BMMCs (**B**, **D**).

Cells were harvested at day 28. Population analysis by light scatter demonstrated one group (Figure 2A,B). The majority of constituents (more than 95%) of both normal and asthmatic rat BMMCs were BC4 (the BC4 antibody reacts with the high-affinity IgE Fc receptor (FcεRI) expressed in rat mast cells) positive as evidenced (Figure 2C,D).

### 2.3. Expression of the Transient Receptor Potential Melastatin 7 (TRPM7) in Rat BMMCs

**Figure 3:**
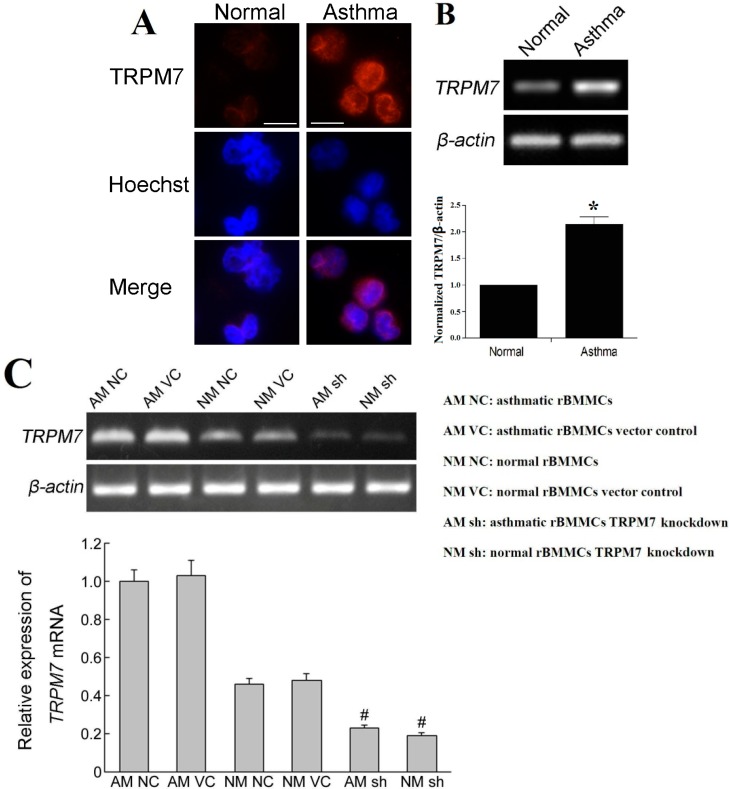
Expression of the transient receptor potential melastatin 7 (TRPM7) in rat BMMCs. (**A**) Immunocytochemistry of TRPM7 in rat BMMCs. Scale bar = 20 µm; (**B**) mRNA expression of *TRPM7* detected by RT-PCR. The mRNA levels of *TRPM7* were significantly increased in asthmatic group compared to the normal group (* *p* < 0.05); (**C**) Expression of *TRPM7* mRNA detected by RT-PCR. The mRNA levels of TRPM7 were significantly decreased in *TRPM7* knockdown groups (^#^ *p* < 0.05).

We consistently observed expression of TRPM7 in both normal and asthmatic rat BMMCs (Figure 3). Immunofluorescence of BMMCs proved that TRPM7 was expressed in both normal and asthmatic rat BMMCs, and the RT-PCR showed that the expression of TRPM7 in asthmatic rat BMMCs was stronger than that in the normal rat BMMCs.

### 2.4. Effects of 2-Aminoethoxydiphenyl Borate (2-APB) on Antigen-Induced Degranulation in Rat BMMCs

**Figure 4:**
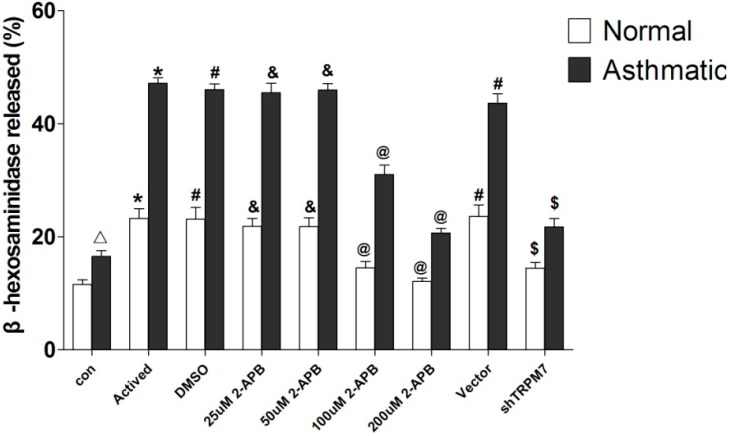
Levels of β-hexosaminidase activity in rat BMMCs. ^Δ^ compared to the normal group, the asthmatic group was significantly higher (*p* < 0.05); * compared to the control group (con), the Activated group was significantly higher (*p* < 0.05); **^#^** compared to the Activated group, the DMSO group and Vector group had no significant change (*p* > 0.05); ^&^ compared to the Activated group, the 25 µM 2-APB group and 50 µM 2-APB group had no significant change (*p* > 0.05); **^@^** compared to Activated group, the 100 µM 2-APB group and 200 µM 2-APB group were significantly decreased (*p* < 0.05); ^$^ compared to the Vector group, the shRNA group was significantly decreased (*p* < 0.05).

The secretion of β-hexosaminidase is the hallmark of an allergic reaction [15]. As shown in Figure 4 and Table 1 (data are means ± SD), the β-hexosaminidase activity was significantly higher in the asthmatic group than in the normal group (*p* < 0.05). When treated with 100 or 200 μmol/L 2-APB, β-hexosaminidase activitives were significantly decreased compared to the activated group (*p* < 0.05), the reductions were more significant in the asthmatic BMMCs (*p* < 0.05).

### 2.5. Effects of 2-APB on Cytokine Secretion in Rat BMMCs

**Figure 5:**
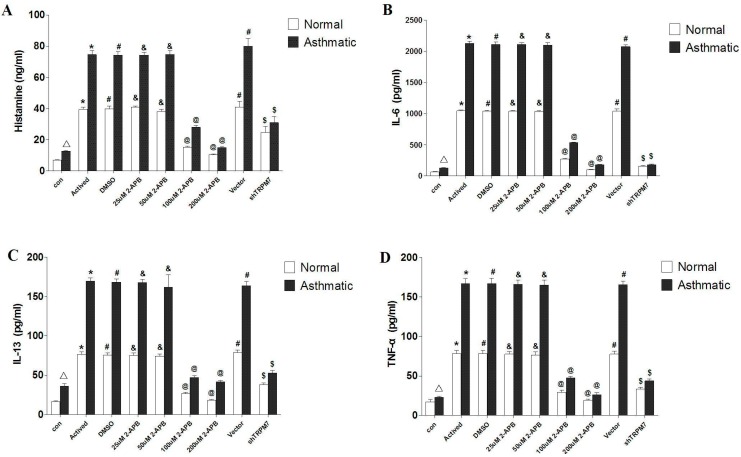
Levels of cytokines in rat BMMCs. (**A**) histamine; (**B**) IL-6; (**C**) IL-13; (**D**) TNF-α. ^Δ^ compared to normal group, the asthmatic group was significantly higher (*p* < 0.05); * compared to the control group (con), the Activated group was significantly higher (*p* < 0.05); **^#^** compared to the Actived group, the DMSO group and Vevtor group had no significant change (*p* > 0.05); ^&^ compared to the Activated group, the 25 µM 2-APB group and 50 µM 2-APB group had no significant change (*p* > 0.05); **^@^** compared to the Activated group, the 100 µM 2-APB group and 200 µM 2-APB group were significantly decreased (*p* < 0.05); ^$^ compared to the Vector group, the shRNA group was significantly decreased (*p* < 0.05).

Table 2 (data are means ± SD) and Figure 5 show the profiles of IL-6, IL-13, TNF-α, histamine in the asthmatic group and the control group. After stimulation of DNP–BSA, concentrations of IL-6, IL-13, TNF-α and histamine were significantly higher in asthmatic activated group than in the normal activated group (*p* < 0.05). When treated with 100 and 200 μmol/L 2-APB, the levels of histamine, IL-6 and IL-13 secretion were significantly decreased (*p* < 0.05) in both asthmatic groups and normal groups; these reductions were more significant in asthmatic BMMCs groups (*p* < 0.05). When treated with 100 μmol/L 2-APB, TNF-α levels were also significantly reduced in both asthmatic groups and normal groups, which were more significant in asthmatic BMMCs groups (*p* < 0.05). When treated with 200 μmol/L 2-APB, TNF-α levels were also significantly reduced in both asthmatic groups and normal groups, but no significant decrease was found between normal and asthmatic BMMCs groups (*p* > 0.05).

### 2.6. Effects of TRPM7 Knockdown on Antigen-Induced Degranulation in Rat BMMCs

Table 1 and Figure 4 show the β-hexosaminidase activity in the *TRPM7*-knockdown BMMCs. After down-regulation of TRPM7, the β-hexosaminidase activity was significantly decreased both in the asthmatic group and the normal group. The β-hexosaminidase activity was significantly lower in asthmatic BMMCs TRPM7 knockdown group compared to the normal BMMCs *TRPM7* knockdown group (*p* < 0.05).

### 2.7. Effects of TRPM7 Knockdown on Cytokine Secretion in Rat BMMCs

The results of IL-6, IL-13, TNF-α, histamine secretion are shown in Table 2 and Figure 5. After stimulation of DNP–BSA, the histamine, IL-6, IL-13 and TNF-α secretion in *TRPM7*-knockdown group were significantly decreased (*p* < 0.05) in both normal and asthmatic BMMCs groups. The levels of IL-13 and TNF-α were significantly lower in asthmatic BMMCs groups than in normal BMMCs groups (*p* < 0.05).

## 3. Discussion

In a previous study, rat BMMCs had been found to be appropriate models for studies on the mucosal mast cells [14,16], which play a key role in the pathogenesis of asthma [17,18]. In our study, it has been found that BMMCs from asthma rat models had many differences compared to normal rats, including higher levels of β-hexosaminidase released and higher cytokines (IL-6, IL-13, TNF-α, histamine) secreted. These variances might be caused by asthma, which results in activation of BMMCs. The secretion of β-hexosaminidase is the hallmark of mast cell degranulation, and histamine, IL-6, IL-13 and TNF-α are typical cytokines secreted after mast cell activation: Histamine is an important mediator in the initiation and the development of antigen-induced airway responses, IL-6 contributes to the pathogenesis of asthma [19], IL-13 is needed for IgE formation and TNF-α is amplified the inflammatory response [20,21,22,23].

TRPM7 is a protein of wide-ranging biological and physiological significance. It is a nonselective cation channel and permeable for many divalent cations such as Zn^2+^, Mg^2+^, Ca^2+^, and Mn^2+^ [24,25]. It is regulated by G-protein coupled receptors (GPCRs) and protein kinase A (PKA) [26] and many studies have demonstrated that TRPM7 is involved in fundamental cellular processes including death, survival, proliferation, apoptosis, magnesium homeostasis, synaptic vesicle fusion, thymopoiesis, and cell adhesion [12,27,28,29]. Recent research has found that TRPM7 is essential for mast cell survival [12]. In the present study, TRPM7 is found in both normal and asthmatic BMMCs, and the expression levels are significantly higher in the asthmatic group compared to normal controls. In order to observe its possible role in BMMCs, two methods were applied to inhibit its function, including the application of ion channel blockers 2-APB and the RNAi technique.

2-APB is considered as a store-operated channel inhibitor and a general blocker of TRP channels, such as the TRPM7 channels. In our previous study, we found that 2-APB inhibits FcεRI-induced degranulation and synthesis of inflammatory cytokines in a mast cell line, RBL-2H3 cells [30]. In this study, we have shown that with the application of 2-APB, the secretion of inflammatory factors were suppressed, suggesting that 2-APB inhibits the activation of BMMCs. To compare the difference between asthmatic rat BMMCs and normal rat BMMCs, the inhibitory effects of the asthmatic group are more obvious because the secretion of cytokines were significantly inhibited.

**Table 1:** Levels of β-hexosaminidase activity in rat BMMCs.

Cells	Control (%)	Actived (%)	DMSO (%)	25 µM 2-APB (%)	50 µM 2-APB (%)	100 µM 2-APB (%)	200 µM 2-APB (%)	Vector (%)	shTRPM7 (%)
Normal BMMCs	11.57 ± 0.81	23.19 ± 1.75	23.09 ± 2.07	21.87 ± 1.36	21.78 ± 1.56	14.47 ± 1.18	12.06 ± 0.64	23.57 ± 2.05	14.45 ± 1.01
Asthmatic BMMCs	16.51 ± 1.02	47.13 ± 0.96	46.00 ± 1.04	45.49 ± 1.66	45.93 ± 1.16	30.99 ± 1.65	20.67 ± 0.81	43.59 ± 1.70	21.75 ± 1.48

**Table 2:** Levels of cytokines secretion in rat BMMCs.

Group	Normal BMMCs				Asthmatic BMMCs			
	Histamine (ng/mL)	IL-6 (pg/mL)	IL-13 (pg/mL)	TNF-α (pg/mL)	Histamine (ng/mL)	IL-6 (pg/mL)	IL-13 (pg/mL)	TNF-α (pg/mL)
Control	6.86 ± 0.15	63.02 ± 5.62	16.39 ± 0.90	17.14 ± 2.84	12.61 ± 0.37	126.92 ± 8.28	35.97 ± 3.44	22.91 ± 1.39
Actived	39.31 ± 1.57	1044.14 ± 13.19	76.54 ± 2.96	78.12 ± 3.81	74.59 ± 2.47	2125.26 ± 31.73	169.70 ± 3.98	166.59 ± 6.55
DMSO	39.64 ± 1.79	1036.76 ± 11.60	75.34 ± 2.87	77.89 ± 4.33	74.06 ± 2.40	2109.19 ± 36.45	168.28 ± 4.03	166.59 ± 7.19
25 µM 2-APB	40.71 ± 0.93	1036.10 ± 15.98	75.21 ± 2.83	77.49 ± 3.27	74.15 ± 1.91	2107.87 ± 28.52	167.79 ± 4.38	166.09 ± 5.44
50 µM 2-APB	37.95 ± 1.54	1031.22 ± 12.27	74.10 ± 2.69	76.36 ± 3.89	74.66 ± 2.41	2096.80 ± 40.91	161.87 ± 16.06	164.93 ± 6.44
100 µM 2-APB	15.18 ± 0.71	271.60 ± 8.62	26.77 ± 1.45	29.43 ± 2.82	27.92 ± 1.13	533.42 ± 7.99	46.99 ± 2.24	47.54 ± 1.92
200 µM 2-APB	10.28 ± 0.54	99.38 ± 7.12	18.01 ± 1.36	19.04 ± 1.30	14.77 ± 0.51	180.81 ± 5.54	41.77 ± 1.73	26.45 ± 2.39
Vector	40.68 ± 3.92	1037.53 ± 35.76	78.63 ± 3.17	77.52 ± 3.62	79.96 ± 4.96	2070.80 ± 30.39	163.75 ± 5.62	165.71 ± 4.48
shTRPM7	24.59 ± 3.83	154.09 ± 11.04	37.90 ± 2.22	33.48 ± 2.26	30.92 ± 3.95	180.98 ± 9.64	52.64 ± 3.23	43.76 ± 2.57

The RNAi technique is a relatively specific means to study TRPM7 function. Here, a lentiviral delivery system was used for the transduction of primary BMMCs. After down-regulation of the TRPM7 channels, the β-hexosaminidase activity and release of typical cytokines such as IL-6, IL-13, TNF-α and histamines were significantly decreased, especially in the asthmatic group. As TRPM7 is an important candidate for calcium influx in non-excitable cells, these inhibitory effects may be caused by suppression of calcium influx after antigen activation in TRPM7-knockdown cells, suggesting TRPM7 is a critical channel not only for the survival of mast cells, but may also be important for their activation.

In this study we also found a significant difference between TNF-α levels of normal and asthmatic BMMCs after application of 100 μM 2-APB. No significant difference was found, however, at a 200 μM 2-APB level. As previously reported, 2-APB at micromole concentrations maximally inhibits the TRPM7 [31]; we suggest here that the inhibition of TNF-α is greatest at the 200 μM 2-APB level.

## 4. Experimental Section

### 4.1. Animals

Sprague-Dawley rats (females) were obtained and maintained in a pathogen-free environment in the facility of the Centre of Animal Experiments of Sun Yat-sen University (Guangzhou, China; Certificate of Conformity: Guangdong Experimental Animal Testing by certificate No. 0101668, 22 February 2012). The rats were housed in a temperature-controlled room with 12-h dark: light cycles, and allowed food and water *ad libitum*. All the experiments described below were performed in accordance with the regulations of the Centre of Animal Experiments of Sun Yat-sen University.

### 4.2. Sensitization and Antigen Challenge

Sprague-Dawley rats were sensitized and challenged with OVA (grade V, A5503; Sigma, St. Louis, MO, USA). They were sensitized on days 0 and 7 by intra-peritoneal injection of 10 mg OVA emulsified in 100 mg of aluminium hydroxide (Guangzhou Chemical Reagent Factory, Guangzhou, China) in a total volume of 1 mL. Seven days after the last sensitization, rats were exposed to OVA aerosol (5% *w*/*v* diluted in sterile physiological saline) for up to 30 min everyday for 2 weeks [32].

### 4.3. Rat Bone Marrow-Derived Mast Cells Culture

Both normal rats and asthmatic rats were euthanized by anesthetization with CO_2_ and cervical dislocation. Bone marrow cells were harvested from the femur by syringe pumping and were separated from the red blood cells using a 70% Percoll (MP Biomedicals, Santa Ana, CA, USA) gradient cushion. Cells were then washed with incomplete IMDM, supplemented with 100 U/mL penicillin/streptomycin (Gibco BRL, Gaithersburg, MD, USA). IMDM (Gibco) supplemented with 5 × 10^−5^ M 2-mercaptoethanol (Gibco), penicillin (100 IU/mL), streptomycin (100 IU/mL), 20% heat-inactivated fetal bovine serum (Gibco), and 10 ng/mL of recombinant rat IL-3 (rRIL-3) (R&D Systems, Minneapolis, MN, USA) (complete IMDM) [33]. Cell numbers were calculated using a hemocytometer. Cultures were re-fed weekly and those containing greater than 95% mast cells at four weeks were used in experiments.

### 4.4. Toluidine Blue Staining

Cells were harvested and plated on lysine-coated glass slides. Cultured cells were fixed in 100% ethanol and dried. Fixed cells were stained with Toluidine blue (Whiga, Guangzhou, China) [33,34].

### 4.5. Flow Cytometry

Phenotypes were analyzed by flow cytometry using FACSCalibur (BD Biosciences, Jan Jose, CA, USA). Suspending cells were harvested from culture plates and washed with PBS by centrifugation. Cell suspensions were incubated with BC4 (BD Pharmingen, San Diego, CA, USA) for 30 min on ice. Cells were washed twice with ice cold PBS and were incubated with goat anti-mouse IgG (H + L) antibody (Invitrogen, Carlsbad, CA, USA) for 1 h. Cells were washed 3 times and then analyzed by flow cytometry.

### 4.6. Detection of TRPM7 Protein in BMMC by Immunofluorescence

BMMCs were fixed in cold acetone (4 °C) for 5 min. After fixation, they were washed in phosphate-buffered saline (PBS; 0.01 mol/L; pH 7.4) and immersed in 0.3% Triton X-100 in PBS. After blocking with 1% bovine serum albumin in 0.01 mol/L PBS for 1 h at room temperature, they were incubated with a goat polyclonal antibody against TRPM7 (Abcam, Cambridgeshire, UK) in PBS containing 3% bovine serum albumin for 24 h (4 °C). After a rinse in PBS at 4 °C, they were labeled with the fluorescein isothiocyanate-coupled donkey anti-goat immunoglobulin G secondary antibody (Jackson Immunoresearch Laboratories, Baltimore, MD, USA) (1:100) 1 h at room temperature. Nuclei were stained with Hoechst stain.

### 4.7. Reverse Transcription-Polymerase Chain Reaction (RT-PCR)

Total RNAs were extracted by using Trizol reagent (Gibco Division of Invitrogen, Carlsbad, CA, USA) according to the manufacturer’s protocol. All procedures were performed with i-cyclor. One gram of total RNA was transcribed with River-Tra Ace (Toyobo, Tokyo, Japan), Oligo (dT) 20, RNase inhibitor, 5-RT buffer, and dNTP mixture. RT-PCR was performed at 42 °C for 20 min and then at 95 °C for 5min using 1.0 g of RNA per reaction. The cDNA was amplified with TaKaRa Taq (TaKaRa, Otsu, Japan), according to the manufacturer’s protocol. The PCR products were separated by electrophoresis using 1.5% agarose gels (sample volume: 10 μL, voltage: 100 V) and visualized by ethidium bromide staining for 10 min and ultraviolet illumination (Kodak, New Haven, CT, USA). The following specific primers were used: TRPM7 (Invitrogen, Carlsbad, CA, USA): 5'-CTGGTCAGAGCACGATGT-3' (sense) and 5'-TGGTATGGATTTGGGTTT-3' (antisense); β-actin (Applied Biosystems, Carlsbad, CA, USA): 5'-TCAGGTCATCACTATCGGCAAT-3' (sense) and 5'-AAAGAAAGGGTGTAAAACGCA-3' (antisense). β-Actin served as an internal control for the efficiency of mRNA isolation and cDNA synthesis.

### 4.8. Design of shRNA against Rat TRPM7

For gene expression, the *TRPM7* gene was PCR amplified and cloned into the multiple cloning site of the pLV vectors (Forevengen, Guangzhou, China). Vectors containing an U6 promoter were used to drive shRNA expression. The shRNA sequence targeting *TRPM7* gene was F: 5'-AACTGGCACCTTTATATCATTAATTCAAGAGATTAATGATATAAAGGTGCCTTTTTTC-3', R: 5'-GAAAAAAGGCACCTTTATATCATTAATCTCTTGAATTAATGATATAAAGGTGCCAGTT-3'.

### 4.9. Generation of Lentivirus Vectors and Transduction of BMMCs

To generate lentivirus vectors, 293T cells in 10-cm culture dishes were cotransfected with 10 μg of pLV vector, 4.8 μg of pGag-Pol, 1.8 μg of pRev, 2.7 µg of pMDG, using lipofectamine 2000 reagent (Invitrogen). Supernatants were collected 48 and 72 h after transfection, filtered through a 0.4-μm membrane, and viruses were concentrated using Amicon Ultra-15 100KD filter. A negative control shRNA carrying by retrovirus vector (VC) was used to verify that the effect seen with *TRPM7*-shRNA was not due to the transfection process.

Infections were carried out in the presence of 10 μg/mL of polybrene. Using pLV vectors with Puromycin marker, cells were selected with 2 μg/mL puromycin following transduction for 1 day for at least one additional day. After removal of the floating cells, the remaining attached cells were analyzed and collected for further experiments.

### 4.10. Stimulation of BMMCs

Two hundred thousand cells per milliliter were seeded per well of 24-well plates and sensitized with 75 μg/L of DNP specific monoclonal IgE (Sigma) overnight. After 3 washes with PIPES buffer (119 mM NaCl, 5 mM KCl, 25 mM PIPES, 5.6 mM glucose, 1 mM CaCl_2_, 0.4 mM MgCl_2_, and 0.1% BSA, pH 7.2), 2-aminoethoxydiphenyl borate (2-APB) (Merck, Munchen, Germany) was added for 60 min at 37 °C when needed. Cells were then stimulated with 20 ng/mL of DNP–BSA (Biosearch Tech, Petaluma, CA, USA) for 30 min at 37 °C and the reaction was stopped by cooling in an ice bath for 10 min [35].

### 4.11. β-Hexosaminidase Assays

β-Hexosaminidase is considered to be a marker of degranulation in mast cells [15,36]. Enzyme assays were performed on cell supernatants and lysates following exocytic stimulation. After stimulation, the cells were centrifuged at 5000× *g* for 1 min, and the supernatants were collected and chilled on ice. The remaining cell pellets were lysed in Modified Tyrode’s buffer (MT: NaCl 137 mM, KCl 2.7 mM, CaCl_2_ 1.8 mM, MgCl_2_ 1 mM, glucose 5.6 mM, Hepes 20 mM, pH 7.4 and BSA 0.1%) containing 0.1% Triton X-100 (the same volume as that used for the supernatant) on ice for 5 min and then centrifuged at 5000× *g*; the supernatant (cell lysate) was collected for enzyme activity analysis. For each well, 50 μL of lysate or supernatant were mixed with 50 μL of reaction buffer (40 mM citrate, pH 4.5) containing 2 mM substrate (4-methylumbelliferyl-*N*-acetyl-β-d-glucosaminide, β-*N*-acetylhexosaminidase substrate, Sigma) in a solid-black 96-well plate and incubated at 37 °C for 15 min. The plate was read on a fluorescence plate reader by using 380-nm excitation and 440-nm emission filters every 5 min five times to obtain an enzymatic rate; analyses were performed in triplicate.

### 4.12. Measurements of Histamine and Cytokines

Histamine, IL-6, IL-13 and TNF-α in the BMMC culture supernatants were measured using ELISA Kits (Histamine, USCN Life Science Inc., Wuhan, China) (IL-6, R&D Systems, Minneapolis, MN, USA) (IL-13 and TNF-α, BOSTER, Wuhan, China) according to the manufacturers’ instructions.

### 4.13. Statistical Analysis

All data are demonstrated as mean ± SD. Differences between groups of data were explored using Independent Samples Test (two-tailed) or analysis of covariance as appropriate. Data were analyzed with the statistical package IBM SPSS 17.0 (IBM Corporation, New York, NY, USA). *p* < 0.05 was regarded as statistically significant.

## 5. Conclusions

In conclusion, our results clearly indicate that inhibition of TRPM7 channels can lead to decreased degranulation and release of cytokines in rat BMMC. Further studies are needed to determine if the same phenomenon is actually involved in the activation process of asthmatic rat lung mast cells *in vivo*. However, our findings suggest that TRPM7 is an important channel and may serve as a potential target for immunotherapy in asthma.
